# Longitudinal changes in the expression of IL-33 and IL-33 regulated genes in relapsing remitting MS

**DOI:** 10.1371/journal.pone.0208755

**Published:** 2018-12-18

**Authors:** Subramaniam Sriram, Guzel Shaginurova, John T. Tossberg, Chandramohan Natarajan, Charles F. Spurlock, Thomas M. Aune

**Affiliations:** 1 Department of Neurology, Vanderbilt University Medical Center, Nashville, TN, United States of America; 2 Department of Medicine, Vanderbilt University Medical Center, Nashville, TN, United States of America; 3 IQuity, Inc., Nashville, TN, United States of America; 4 Department of Pathology, Microbiology & Immunology, Vanderbilt University Medical Center, Nashville, TN, United States of America; Center for Disease control and Prevention, UNITED STATES

## Abstract

**Objective:**

We tested the hypothesis that the expression of IL-33 in MS is dynamic and is likely to reflect the clinical and radiological changes during the course of RRMS.

**Methods:**

MS with either clinical or radiological relapses were recruited for the study and followed for one year. IL-33 and a panel of genes was measured by q PCR and flow cytometry at different time points.

**Results:**

Among 22 RRMS patients, 4 patients showed highest levels of IL-33 at the time they were recruited to the study (Month 0); in 14 patients highest levels of IL-33 were seen between 6–11 months after relapse and in 4 patients maximal levels of IL-33 were seen 12 months after relapse. A similar pattern of IL-33 kinetics was seen when IL-33 was measured by flow cytometry in an additional cohort of 12 patients. The timing of the improvement clinically did not correlate with IL-33 expression with highest expression levels either preceding or following clinical recovery. From our whole genome RNA-sequencing data we found a strong correlation between expression levels of IL-33 and a ~2000 mRNA genes. However, none of these genes encoded proteins involved in either innate or adaptive immunity. Rather, many of the genes that correlated highly with IL-33 encoded to proteins involved in DNA repair or mitochondrial function and mRNA splicing pathways.

**Interpretation:**

Given the neuro-reparative and remodeling functions attributed to IL-33, it is likely that some of the novel genes we have uncovered may be involved in repair and recovery of the CNS in MS.

## Introduction

Onset of new clinical signs consistent with lesions of the optic nerve, spinal cord and brain stem of subcortical white matter represent common presentations of an acute clinical relapse in Multiple Sclerosis (MS) [1]. Dissemination of lesions is space and time is a requisite to make the diagnosis of clinically definite MS [2]. Patients who present with initial neurological symptoms which are typical of MS, but without additional evidence of involvement of lesions in either space or time, are referred to as having Clinically Isolated Syndrome (CIS). Radiologically, relapses are associated with the development of new gadolinium enhancing lesions or new T2 lesions [3,4,5]. Inflammation in the brain is often clinically silent and presence of many new enhancing lesions is not often associated with new clinical signs or symptoms. All relapses (clinical and radiological) eventually resolve with improvements in the clinical disability scores and in the extent and severity of CNS inflammation [6]. Recovery is usually seen in 6–9 months but can sometimes take up to one year. The process of recovery is variable both within each patient in response to relapses at different times and between RRMS patients [7].

Pathologically, the inflammatory signature of the demyelinating lesion consists of a T cell infiltrate, activated macrophages and presence of pro-inflammatory cytokines [2,8]. The prevailing opinion is that the inflammatory changes seen in the brain are the outcome of an autoimmune response directed against an as yet undetermined antigen. The biochemical characteristics of the inflammatory lesions has suggested the activation of both the innate and adaptive arms of the immune system [8,9]. The sequelae of the inflammatory lesions and their evolution is sometimes manifested in the peripheral immune system. We have suggested the expression of novel genes which are likely to indicate the onset of MS and therefore represent a potential biomarker for MS. However, there are currently no immune signatures present in the peripheral immune system which indicates either the activation or resolution of CNS inflammation [9,10]. There are no biomarkers which predict the recovery process following relapses in MS.

In a cross sectional study of RRMS patients, we showed that IL-33 levels were increased in peripheral blood mononuclear cells (PBMC) of RRMS when compared to controls [11]. IL-33 belongs to the IL-1 family of pro-inflammatory cytokines involved in innate immunity and is regarded as an “alarmin” alerting the host of the presence of a pathogen [12,13,14]. As with other members of “alarmins”, IL-33 has dual functions depending on its location. IL-33 present in the nucleus is thought to regulate tissue modelling and repair, while IL-33 which is released extracellularly induces a sterile inflammation [15]. In animal models of CNS demyelination, IL-33 was expressed intracellularly in regions undergoing remyelination and a protective effect of IL-33 has been described in animals following experimental injury to the CNS.

IL-33 expression is regulated by the state of acetylation and deacetylation of lysine residues in the amino terminal tails of histones in the nucleus [16,17,18]. Inhibition of class I family of HDAC’s by Trichostatin A reduced the expression of IL-33 *in vitro* and *in vivo*. In particular we have found a higher expression of HDAC3, one of the members of Class I family of HDAC’s, to be increased in MS patients and its expression was associated with IL-33.

Prior studies had suggested an immuno-protective role of IL-33 following CNS injury. IL-33 is recognized as one of the “alarmins” and thought to play a role in host defense [19]. IL-33 is present in the gut, mucosa and bronchial cells acting as sentinels to detect danger. IL-33 is also expressed at high levels in the CNS and mostly in oligodendrocytes. Since the CNS is not a portal of entry for pathogens, the role of IL-33 in the CNS, is not known [13,20]. However, there are a number of observations which suggest a neuro-reparative role for IL-33 in the CNS. In vivo administration of IL-33 reduces the severity of experimental allergic encephalitis (EAE), an animal model of inflammatory demyelination with similarities to human multiple sclerosis when given after the development of paralytic signs [21]. Also, in vivo administration of IL-33 improves the myelin content in regions of spinal cord following spinal cord trauma [20] [22]. We have shown that following induction of demyelination with lysolecithin, in vivo treatment of animals with Poly-IC, (known inducer of IL-33) showed increased expression of IL-33 in the remyelinating regions in brain and enhanced remyelination [23]. In vivo treatment with IL-33 improves outcome after stroke and viral infections [24].

For these reasons, we proposed that the systemic expression of IL-33 may reflect a response to recovery from inflammatory injury [25,26]. Our current study was aimed at examining the changes in the expression of IL-33, genes which are associated with IL-33 expression and HDAC3/HDAC1 in MS patients following a relapse.

## Materials and methods

### Human subjects

Blood for the studies were obtained either from the Accelerated Cure Project (ACP) or from the MS clinic at Vanderbilt University Medical Center. Patients recruited for the study were designated to have a relapse when they showed new neurologic symptoms which were corroborated by changes in the clinical exam with a change of 1.0 step in the EDSS, the development of a new enhancing lesion in brain or spinal cord or a new T2 lesion following MR imaging. Patients were recruited to the study within 12 weeks of the onset of clinical symptoms. If the symptoms were present for greater than 8 weeks or the onset was uncertain, the presence of new enhancing lesions was required for entry. Blood for the studies were drawn at month 0 and between months 5–7, 8–11, and 12–15 after a confirmed relapse. If the patient received corticosteroids, the timing of the first blood draw was done between 28–42 days after receiving corticosteroids. Neurological examinations were done between month 5–7, 8–11 and 12–15 after relapse. MRI studies were done between 12–15 months after entry into study. Relevant institutional review board approval from all participating sites was obtained. EDSS scores were tabulated by the examiner in an unblinded manner. Our other neurologic disease (OND) cohort of patients was also recruited from the Neurology clinic at Vanderbilt University Medical Center. The OND cohort consisted of 2 patients with neurosarcoidosis, 2 patients with non-inflammatory optic neuritis, 2 patients with NMO, two patients with spondylitic myelopathy and one patient with CNS vasculitis. 37 RRMS patients 10 OND patients were recruited from Vanderbilt Medical Center. 42 samples from patients who presented with clinically isolated syndrome obtained from the ACP. A biomarker profile of the CIS patients has been published before [27].(Summarized in S2 Table)

The clinical study was approved by the Institutional Review Board of Vanderbilt Medical Center under two applications: IRB#160091, “Regulation of IL-33 in RRMS” and IRB #161663 Biomarkers and regulation of IL-33 in MS. All of the consent obtained was informed, written and documented. There was no waiver of any of the individuals from the consenting process.

For patients whose blood sample was obtained from ACP, the IRB approval was obtained by the Accelerated Cure project who shared their clinical material with us.

### mRNA transcript determination

*IL-33* data for CIS patients was obtained using TaqMan low density arrays. We improved our assays and subsequent studies were done as follows: Total RNA was purified from Paxgene tubes using PreAnalytix kits according to standard protocols. cDNA was synthesized using Superscript III with oligo-dT as primer (Invitrogen, Carlsbad, CA, USA). Quantitative PCR measurements were made using a QuantStudio 12K Flex Real-time PCR system (Applied Biosystems). Primer pairs were designed for *IL-33*, *HDAC1*, *HDAC3* and *HDAC5 (S1 Table)*. These targets were measured using SYBR green chemistry according to the manufacturer’s protocol (PowerUp SYBR Green Master Mix, Applied Biosystems). A TaqMan Low Density Array (TLDA, Applied Biosystems) was designed to analyze expression levels of target genes including ‘housekeeping genes’ in 300ng cDNA. Gene probes included on the TLDA plate were: *GAPDH*, *DND1*, *GABPB1-AS1*, *GPR160*, *LPAR6*, *PET100*, *LILRA5*, *RNF208*, and *SERTAD3*. Relative expression levels were determined directly from the observed threshold cycle (Ct). Expression levels were normalized to *GAPDH* according to the formula, 2^(GAPDH Ct–TARGET Ct)^ [28]. The list of genes analyzed are shown in S3 Table.

Analysis of differentially expressed mRNA genes were determined by whole genome RNA-seq using total RNA from Paxgene Blood RNA tubes treated with DNAse I using standard protocols (Qiagen/Preanalytix). Poly(A)+ Tru-Seq Stranded kits (Illumina) were used to prepare sequencing libraries. Sequencing experiments were performed using an Illumina HiSeq 2500 instrument generating 100bp, paired-end reads with an average read depth per sample of 35 million reads. Sequencing was performed using healthy control subjects with no family history of autoimmune disease and no chronic or acute infections (n = 8); subjects at the time of a clinically isolated syndrome who later developed clinically definite relapsing remitting multiple sclerosis MS-C (n = 6); RRMS patients at the time of diagnosis but prior to initiation of therapy MS-N (n = 6); and established RRMS patients with 1–3 years’ duration of disease, MS-E (n = 6). MS-E subjects were not on disease-modifying therapies. Mapping to GRCH37/hg19 was performed using established pipelines (1) Expression of long non-coding RNAs in autoimmunity and linkage to enhancer function and autoimmune disease risk genetic variants and (2) Defective structural RNA processing in relapsing-remitting multiple sclerosis]. 12,850 mRNAs were expressed with an average FPKM in MS >0.5 FPKM. Differentially expressed mRNAs from this reduced list were determined using DESeq2 [29]. 48-well TaqMan Low-Density Array Cards were created by selecting the 46 top mRNAs that exhibit maximal log2 fold change difference and lowest q-value in MS versus healthy control.

### Statistical analysis

Longitudinal data were assessed comparing the indicated time points using the Wilcoxon paired statistical test. P-values for correlation studies were determined by linear regression using GraphPad Prism Software. Pathways analysis p-values were determined using GREAT Analysis [30]. The Welch's corrected T-test not assuming equal variances was used to calculate P values in two-way comparisons.

### Flow cytometric analysis:

Peripheral blood mononuclear cells (PBMCs) were separated from patients’ blood, a day before the flow cytometry analysis, following Histopaque-1077 (Sigma, St. Louis, MO) gradient centrifugation method. Collected PBMCs were plated in 60 mm cell culture dishes in RPMI-1640 medium containing 10% FBS. The next day, cells were harvested and washed twice with ice cold PBS at 2500 rpm for 10 min each time. Part of the PBMCs (2.0 X 10^6^) were incubated with FITC conjugated mouse anti-human CD14 antibodies (BD Biosciences; Cat 555397, San Diego, CA) in 150 μl of 5% BSA containing PBS, on ice for 1 h. Cells (both unstained and CD14 stained) were then washes twice with ice cold PBS, vortexed briefly to loosen the centrifuged cells and fixed with 250 μl of BD cytofix/perm solution (BD Biosciences, San Diego, CA) for 20 min at 4°C. Cells were then washed twice with 0.75 ml of ice-cold perm/wash solution (BD Biosciences, San Diego, CA) at 3000 rpm for 5 min each. After washing, cells were incubated with 3 μl of PE conjugated rat (IgG2B) anti-human IL-33 (R&D systems; cat # IC3625P; Minneapolis, MN) antibodies in 150 μl volume of perm wash buffer at room temperature for 1 h. Part of the unstained and CD14 stained cells were also stained with rat (IgG2B) isotype control antibodies (R&D systems; cat # IC013P; Minneapolis, MN) in 150 μl volume of perm wash buffer at room temperature for 1 h. Cells were then washed twice with 0.75 ml of ice-cold perm/wash solution and re-suspended in 0.5 ml of PBS for flow cytometry analysis. S1 Fig shows the flow cytometric distribution of CD14+ IL-33+ cells. Data were quantitatively analyzed using Flow Jo software and expressed as percent positive cells. IL-33 positive cells in the CD14+ and CD14 negative populations were calculated by subtracting isotype matched controls.

## Results

### Expression of IL-33 in relation to timing of Clinically Isolated syndrome (CIS)

**Fig 1:**
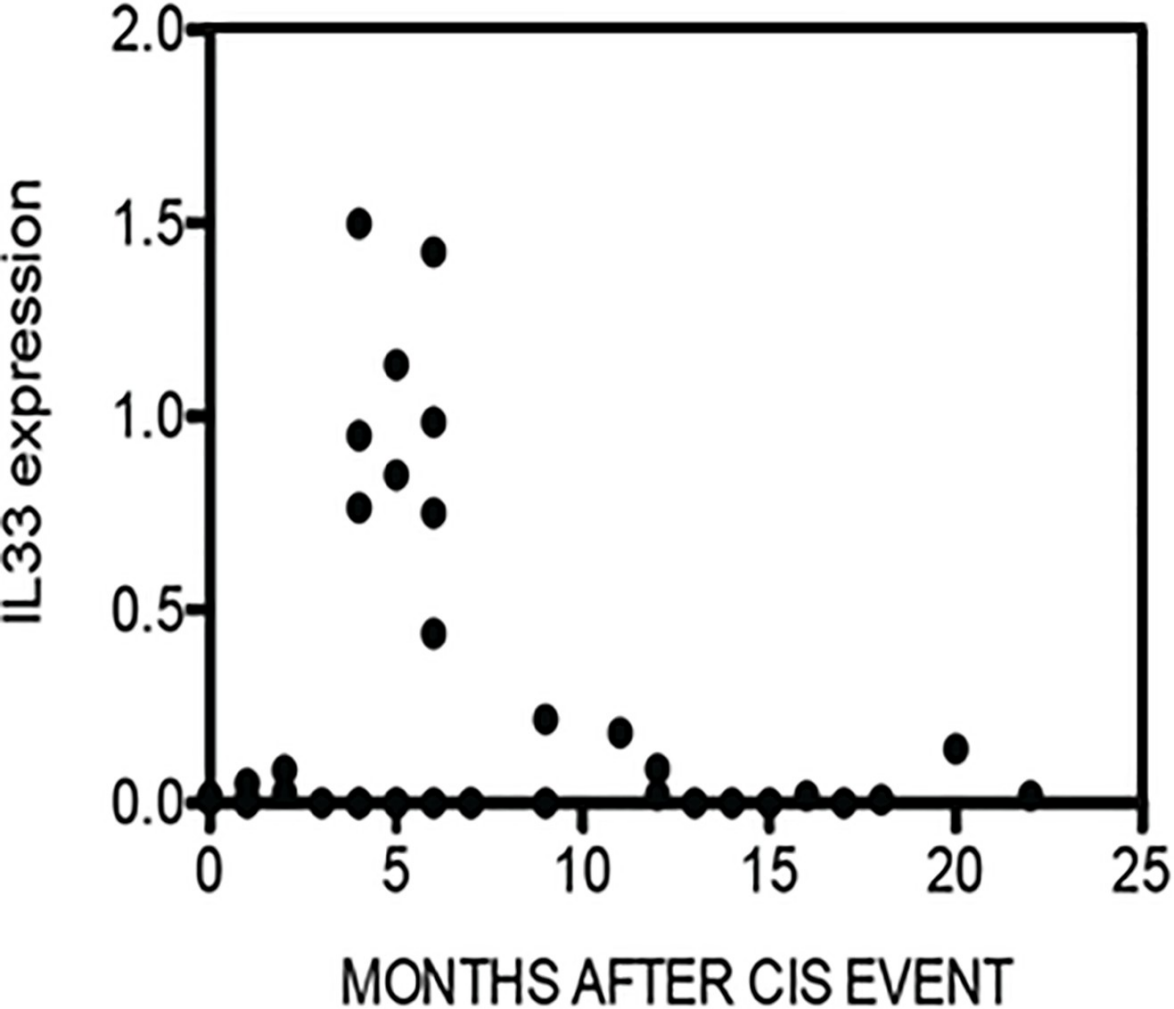
Cross sectional analysis of IL- 33 expression after an initial event CIS event in subjects who were eventually diagnosed with RRMS. IL-33 expression levels were determined by PCR and normalized to expression levels of GAPDH. *The X-axis indicates months after CIS event in a cross-sectional study*, *N = 39*. *Each black dot represents the IL-33 expression in an individual patient and plotted against the time of blood draw to the date of the first clinical event of MS*.

To explore dynamic changes in IL-33 expression during evolution of MS, we examined IL-33 expression in relation to the time of the blood draw after the initial CIS event using data previously obtained [31]. *The data on the expression of IL-33 in the cohort of CIS patients have not been previously published*. We found that IL-33 expression levels were not uniform across the population of CIS patients. Rather, IL-33 levels were relatively low in samples obtained 0–3 months after the initial CIS event, increased 4–6 months after the initial CIS event, and progressively declined from 8–22 months (Fig 1).

### Longitudinal expression of IL-33 in RRMS

We next examined the changes in the expression of IL-33 in patients recovering *from a relapse of MS*. Details of the patient profiles are shown in Table 1. Twenty five patients were recruited and 22 completed the study and their blood was subjected to analysis. There were 13 women and 9 men with a mean age of 34.9 years. Twelve patients had new enhancing lesions in either brain or cervical spine along with clinical signs consistent with changes in exam from earlier records. Three patients had showed an increase in T2 lesion size or number. Six patients had no changes on MRI but worsened in their disability scores by more than one step on the EDSS from their prior visit.

**Table 1:** Clinical features of RRMS patients.

Pt ID#	Gender /Age	Onset of Sx	MRI	Exam at Month 0	Steroids	DMD prior to relapse	DMD after relapse	Exam at M 12–15	MRI at M 12–15
Group 1									
8	M/39	4 weeks	New enhancing lesions brain	Left leg weakness	Yes	Fing	n	Unchanged	Resolution of enhancing lesions
10	F/22	3 weeks	Unchanged from prior exams	Numbness of left arm	No	Nat	Nat	Normal sensory exam	Unchanged from prior studies
17	M/38	Uncertain	Unchanged from prior exam	Decreased sensation in arms and leg	No	None	GA	Decreased sensation arm and leg	Unchanged from prior studies
19	F/34	6 week	New T2 lesion in brain and C spine	Ataxia of gait	No	Nat	Nat	Unchanged	T2 lesions decreased in size and number
Group 2									
3	M/43	8 weeks	New T2 lesion C spine	Decreased sensation hands and feet, new onset Lhermittes sign	No	Nat	Nat	Unchanged from month 6	Unchanged, no new lesions
4	F/38	1 week	New enhancing lesion brain	Mild numbness on left side	Yes	Ter	Ter	Normal exam	MRI lesion resolved
5	F/34	1 week	New enhancing lesion brain	Unchanged from prior exam	No	B-IFN	Fing	New onset left side numbness	New enhancing lesion brain
9	F/32	8–12 weeks	New enhancing lesions brain	Bilateral leg weakness and ataxia	Yes	GA	Fing	Exam Unchanged	Not done
12	F/24	8 weeks	Multiple new enhancing lesions	Ataxia of gait	Yes	None	B-IFN/Nat	Exam Unchanged	Stable T2 lesions
15	M/40	1 week	Unchanged from prior exam	Drags left leg	Yes	Nat	Nat	Exam Unchanged	Not done
Group 3									
7	F/23	12 weeks	MRI brain and C spine unchanged from earlier exams	Decreased sensation in hands	No	B-IFN	Tec	Sensory changes improved	MRI stable from base line
6	M/36	12 weeks	Enhancing lesion brain and cervical cord	Decreased sensation in hands	No	GA	GA /Nat month 11	Unchanged	At month 11 developed new lesions—switched to Nat
1	F/33	4 weeks	New lesion in left internal capsule	Rt sided weakness and ataxia	Yes	B-IFN	B-IFN	Rt side weakness resolved	MRI lesion brain resolved
2	M/45	2 weeks	Unchanged from prior exam	Rt leg ataxia and weakness	Yes	GA	Nat	Exam unchanged	No change
14	F/23	1 week	Multiple new enhancing lesions	Bilateral upgoing toes	Yes	Tec	Nat	Normal exam	No new enhancing lesions
20	M/47	6 weeks	Enhancing lesion cervical spine MRI	Ataxia of left hand leg, gait requiring cane	Yes	Stopped Fing	None	Normal exam	Not done
21	M/22	1 week	New lesion spinal cord	Weakness of left arm and leg	Yes	None	Nat	Mild loss of sensation Rt Arm	C spine MRI lesion has resolved
22	F/47	4 weeks	Multiple new T2 lesions	Decreased visual acuity	Yes	None	GA	No return of vision	Unchanged
Group 4									
11	F/29	8 weeks	Multiple new enhancing lesions	Ataxia of gait	Yes	Nat/poor compliance)	Nat	Normal exam	Not done
13	F/46	5 weeks	MRI unchanged from prior exam	Decreased sensation right arm and leg	No	None/poor compliance	GA	Unchanged from M6	Not done
16	M/31	Uncertain	New enhancing lesion spine	Decreased sensation in hands	no	Fing	Fing	Improvement in sensations	Enhancing lesion C spine resolved
18	F/42	4 weeks	Enhancing lesion brain	Homonymous hemianopsia	No	None	B-IFN	Visual symptoms persist	Enhancing lesion brain resolved

B-IFN = beta interferon; Fing = Fingolimod; Nat- = Natalizumab; Ter = Teriflunomide; GA = Glatiramer Acetate; Tec = Tecfidera

Twelve patients received methylprednisone (iv) 1gm daily for 3–5 days or oral dexamethasone 80mg daily for 5 days. Eight patients were not on any therapy for MS at the time they entered the study; 5 patients were drug naïve, 3 patients were non-compliant with their DMT for 6 months or more at the time of the relapse. Of the remaining 14 patients, 3 were on Natalizumab, 3 on Fingolimod, 2 on Beta Interferon, 3 on Glatiramer Acetate, 2 on Tecfidera and one on Teriflunomide. During the period of the study, 9 patients were on Natalizumab, 2 on beta interferon, 4 on Fingolimod, 2 on glatiramer acetate, 2 on Tecfidera, 1 patient on Teriflunomide and one patient stopped his therapy with Fingolimod after being on it for 3 months and was on no disease modifying treatment for the rest of the study period.

At the end of 12 months, with the exception of three patients who showed persistence of their original symptoms, the rest had improved and returned to baseline disability scores which were documented 6 months to 1 year prior to their relapse. In all patients who had enhancing lesions, the inflammatory activity had resolved by 12 months. In 7 patients the MRI lesions were stable and in one the T2 lesion had decreased in size. Two patients showed new inflammatory lesions at month 12. One patient was symptomatic from the lesion while the new active lesion was clinically silent in the other patient. Clinically, 17 patients improved from their initial presenting symptoms at month 0. In four patients symptoms were persistent and in one patient a new symptom was associated with new lesion activity.

**Fig 2:**
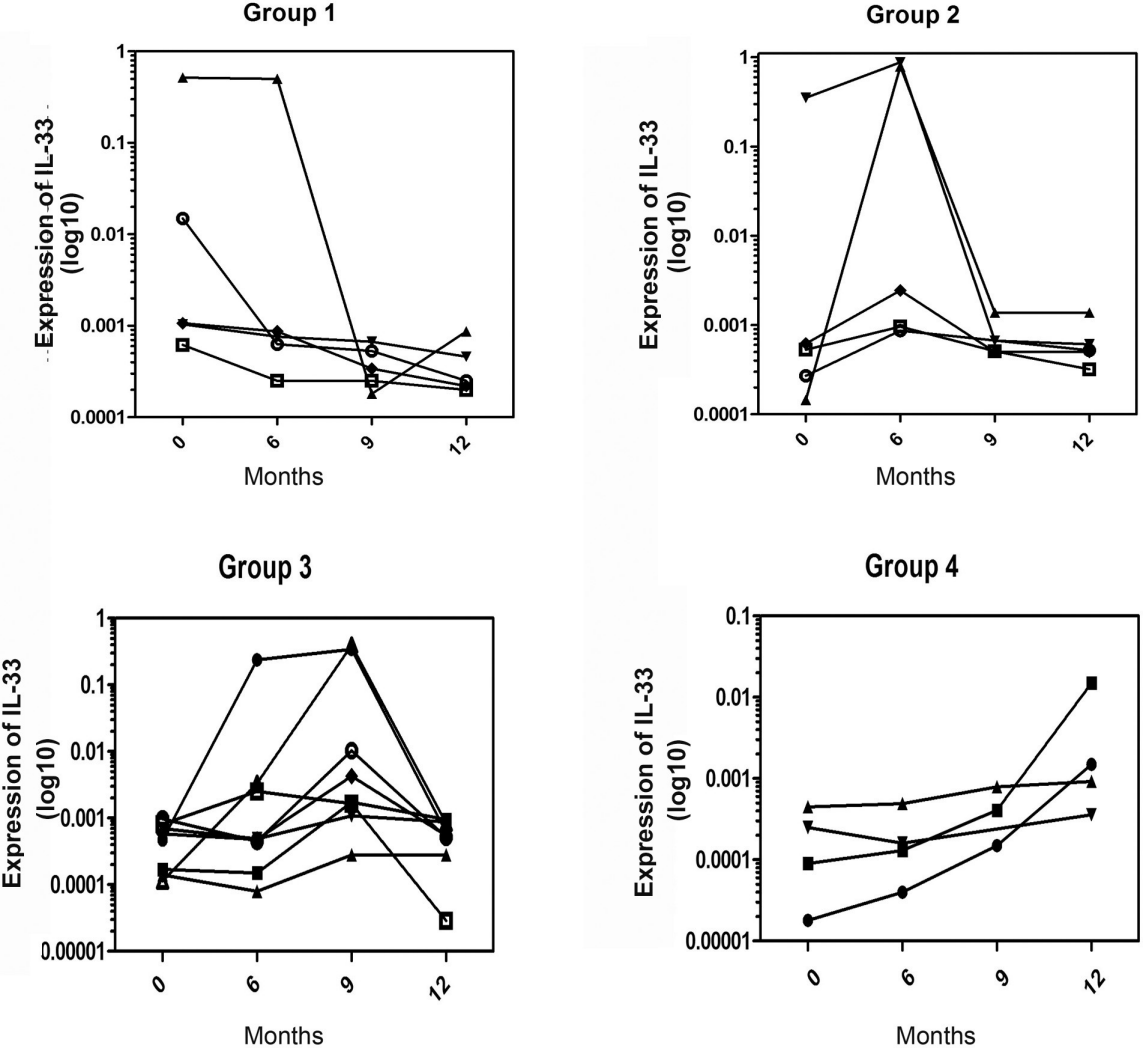
(a-d) Longitudinal analysis of expression of IL-33 in each individual RRMS patients following a radiological or clinical relapse. All results are expressed as Ct values for IL-33 normalized to GAPDH. Results are shown in four different epochs. Group 1 = maximal Ct value for IL-33 at month 0, Group 2, maximal Ct value between months 5–7, group 3, maximal ct value between month 9–11 and group 4, maximal Ct value after month 12. (P, 0.01 for maximal Ct values for group 1, 2 and 3 compared to month 12–15; for group 4, (p, 0.01 for month 12–15 compared to month 0).

**Fig 3:**
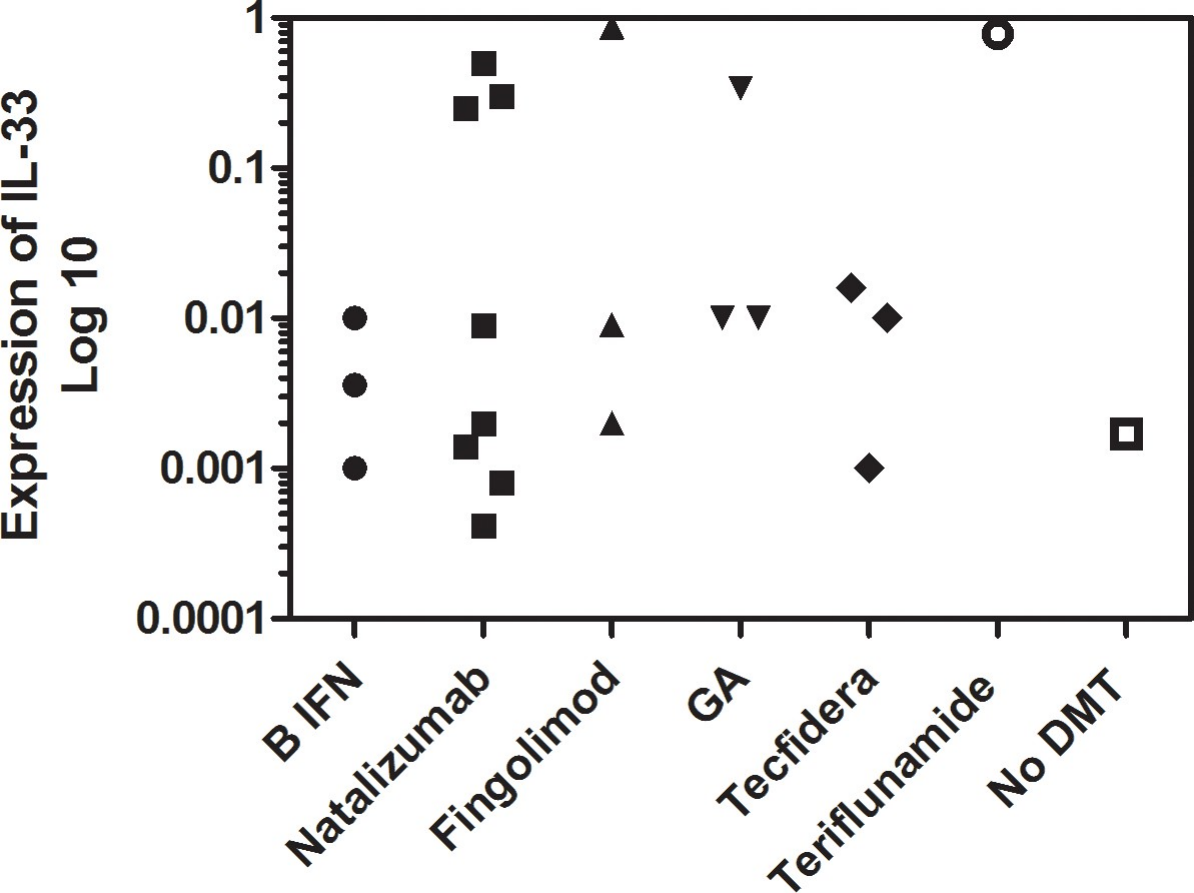
The maximal expression of IL-33 in MS patients on different disease modifying therapies (DMT). The Y axis shows the different therapies patients were maintained on during the 12 month period after a relapse. Each symbol is the maximal IL-33 expression of an individual patient on a specified DMT.

We stratified the dynamic changes in IL-33 into four major time periods (Group 1-Group IV) according to the highest expression of IL-33 (Ct value normalized to GAPDH) seen during the course of the study: Group 1, month 0, 4 patients, Group 2, month 5–7, 7 patients, Group 3, month 8–11, 8 patients, group 4, month 12–15, 4 patients. In groups 1–3, the highest level of expression of IL-33 (month 0 for group 1, month 5–7 for group 2 and month 8–11 for group 3) was statistically greater when compared to month 12 (P,0.02 for all groups). In group 4, highest levels of expression of IL-33 was seen at months 12–15 and the expression was not statistically greater when compared to either month 0 or month 5–7. (Fig 2, Table 2). There was no correlation between the changes in the IL-33 expression and the disease modifying therapy the patients had received. Although the expression of IL-33 was higher in 8 patients receiving Natalizumab, it was not statistically different when compared with other DMT’s (Fig 3).

**Table 2:** Expression of IL-33 (Ct values normalized to GAPDH) in RRMS patients. (Bolded numbers represent the group of patients who had the highest expression of IL-33 during the times after relapse).

Group 1 Pt #	0	5–7 month	8–10 moth	12–15 months
8	**0.00107**	0.00087	0.00034	0.00022
10	**0.518**	0.5001	0.00018	0.00087
17	**0.00104**	0.00076	0.00067	0.00046
19	**0.0149**	0.00063	0.00053	0.00025
Group 2				
3	0.00062	**0.0025**	0.0025	0.0002
4	0.000145	**0.78731**	0.00138	0.00138
5	0.35111	**0.87661**	0.00067	0.00061
9	0.00062	**0.00245**	0.0005	0.0005
12	0.00027	**0.00087**	ND	0.00052
15	0.00053	**0.00096**	0.00051	0.00032
Group 3				
7	0.00081	0.00252	**0.00165**	0.00029
6	0.00047	0.2376	**0.34376**	0.00061
1	0.00017	0.00015	**0.00173**	0.00096
2	0.000114	0.003331	**0.39502**	0.00084
14	0.00014	0.00008	**0.00026**	0.00026
20	0.0007	0.00049	**0.00107**	0.00088
21	0.00058	0.00048	**0.00426**	0.00052
2	0.00098	0.00045	**0.01031**	0.00052
Group 4				
11	0.000018	0.00004	0.00015	**0.0015**
13	0.00009	0.00013	0.00041	**0.01494**
17	0.00045	0.00049	0.00079	**0.00092**
	0.00025	0.00016	ND	**0.00036**

In order to determine the changes in the protein expression of IL-33 in PBMC, we followed RRMS patients after relapse and determined the changes in the expression of IL-33 using flow cytometry at 0 month, 6–9 month and 12–14 month after relapse (Table 3 and Table 4). We prospectively recruited 14 RRMS who were switched to either Tecfidera or Beta IFN (any formulation) after the relapse. Two patients were lost in follow up. There were 7 patients on Tecfidera and 5 on Beta IFN. In two patients flow cytometry was not done at all time points. Three patterns of changes were seen in the course of one year.

**Table 3:** Cohort of RRMS patients after relapse treated with either Tecfidera or Beta Interferon who underwent flow cytometry.

Pt ID	Age-Gender	MRI M0	Exam at M0	DMD prior	DMD after relapse	Follow up M12
001	23/M	New lesion corona radiata	Ataxic gait and weakness	Non compliant	Tec	Improved at M6
002	33/M	Lesion cervical cord	Left sided weakness	B-IFN	B-IFN	Resolved
003	39/M	5 new T2 lesions	Normal	B-IFN	Tecfidera	No new MR lesions
004	51/F	Unchanged from prior	Worsening visual acuity	B-IFN	Tecfidera	Visual acuity to baseline
005	31/F	Two new enhancing lesions	Unchanged from prior visit	New Dx RRMS	Tecfidera	MR lesions resolved
006	39/F	Two new enhancing lesions	Gait ataxia and paretic bladder	Non Compliant	B-IFN/Tec	Developed new lesions at M12
007	50/F	Three enhancing lesions brain	Left leg numbness	New Dx RRMS	B- IFN	MRI and sensory lesions resolved
008	48/F	Increase in T2 lesions	Mild ataxia	New Dx RRMS	B-IFN	MRI and exam stable
009	38/F	New T2 lesion in brain stem	Weakness in arm and leg	No DMD	B-IFN	Lesion Resolved
010	38/F	No change from prior scan	Worsening visual acuity,	B-IFN	B-IFN	Visual acuity to base line
011	33/F	New lesion T spine	Numbness in legs	New Dx RRMS	Tecfidera	T spine lesion resolved
012	46/F	Increase in size of plaque in left hemisphere	No change	Tecfidera	Tecfidera	No new lesions noted

**Table 4:** Flow cytometric study on the expression of IL-33 in CD14+ and CD14- population of PBMC at different time points after relapse.

PT #		0 Month			M 6–9			M 12–14		
		CD14+/	CD14-/	Total IL-33	CD14+/	CD14-/	Total IL-33	CD14+/	CD14-/	Total IL-33
		IL33+ (%)	IL33+ (%)		IL33+ (%)	IL33+ (%)		IL33+ (%)	IL33+ (%)	
1	CD14&IL33	0.02	0	0.02	7.75	0.16	7.91	ND	ND	ND
2	CD14&IL33	5.61	1.32	6.93	5.17	0.08	5.25	2.08	0	2.08
3	CD14&IL33	1.05	0.52	1.57	0.15	0.05	0.2	5.89	0.09	5.98
4	CD14&IL33	1.74	2.3	4.04	ND	ND	-	0.14	0.94	1.08
5	CD14&IL33	0.85	0	0.85	2.09	0.64	2.73	0.75	0.62	1.37
6	CD14&IL33	1.04	0.18	1.22	1.62	0.43	2.05	2.63	1.08	3.71
7	CD14&IL33	0.7	0.6	1.3	9.83	0.36	10.19	1.43	0.04	1.47
8	CD14&IL33	2.38	0	2.38	0.63	0.12	0.75	3.02	0.38	1.34
9	CD14&IL33	3.86	1.1	4.96	0.43	0	0.43	2.26	0.1	2.36
10	CD14&IL33	0.08	1.71	1.79	0.52	0.02	0.54	1.59	0.03	1.62
11	CD14&IL33	1.66	0	1.66	13.72	2.01	15.73	2.49	0.22	2.71
12	CD14&IL33	0.76	0.07	0.83	1	0	1	1.37	0.01	1.38

**Fig 4:**
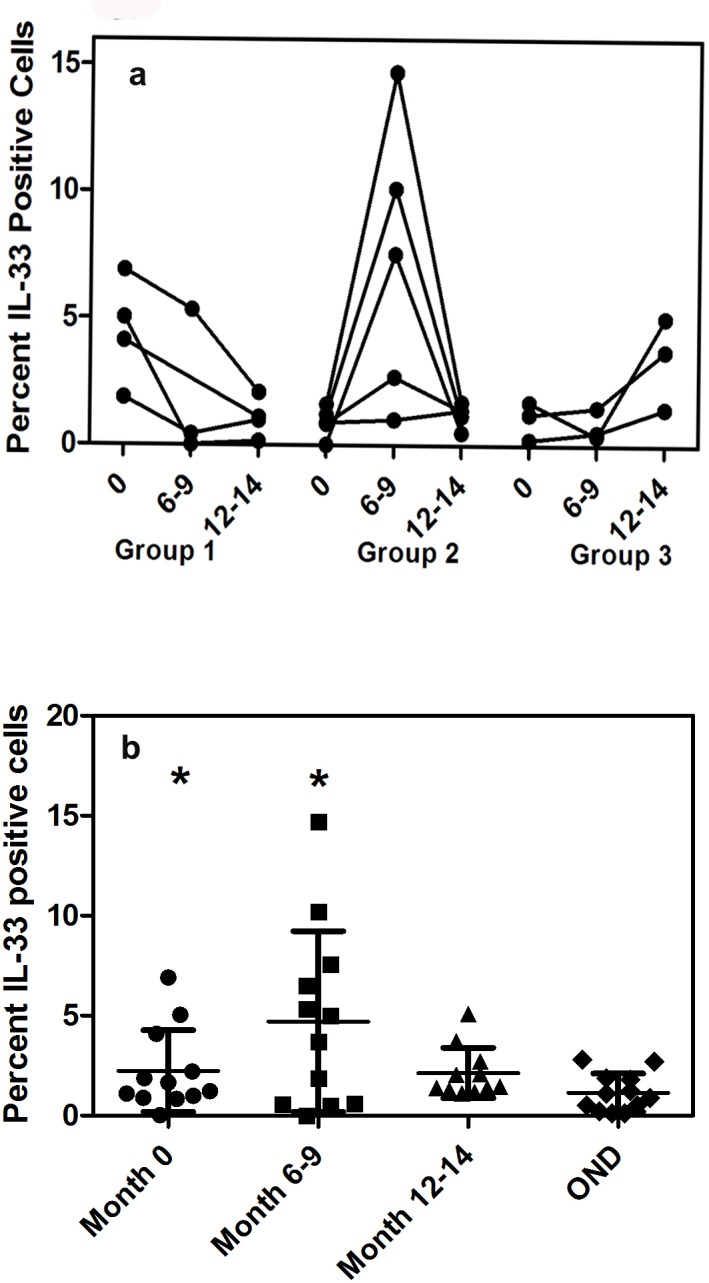
4a-b Flow cytometric analysis of changes in IL-33 expression in PBMC after relapse. (a) Profile of IL-33 in patients showing highest level of expression of IL-33 at month 0, month 6–9 and month 12–15 (b) cross sectional analysis of IL-33 levels between MS patients after relapse and in OND patients.

In four patients, the highest expression of IL-33 was seen when the flow cytometry studies were done at the time the relapse was ascertained and within 1 month of onset of symptoms. In this group of 4 patients (patient #, 2, #4, #9 and # 10), 4.48±2.01% of CD14+ and CD14- lymphocytes expressed IL-33 soon after relapse. At month 12 the number of IL-33+ cells decreased to 1.08±0.7% (p = 0.05 month 0 compared to month 12–15). In 4 patients (#1, #5, #7,and #11) the increase was seen midway through the course of the recovery. There were 1.11±0.8% lymphocytes that expressed IL-33 at time point 0. At 6–9 months after relapse the mean number of IL-33 positive cells increased to 5.4 +/- 4.4% and at 12 months it had decreased to 2.4±1.5% (p = 0.02, 0 month versus 6–9 month). There was no statistical difference in the expression between 0 month and 12–14 month, group 3. In 4 patients (#3, #6, #8 and #12) the increase was maximal at month 12 when compared to either month 0 or month 6–9 (Fig 4A). There was no statistical difference in the IL-33 expression between the different time points in this group. To examine the constitutive expression of IL-33 in a control cohort, we examined the IL-33 expression in 10 OND and compared the expression of IL-33 at month 0 and months 6–9. In the OND cohort the mean expression of IL-33 CD14+ lymphocytes was 1.7+/-1.3%. In a one way analysis of ANOVA there was significant difference in the variance between MS patients after relapse and the OND population (Fig 4B, P<0.02).

### Longitudinal analysis of HDAC3 expression

**Fig 5:**
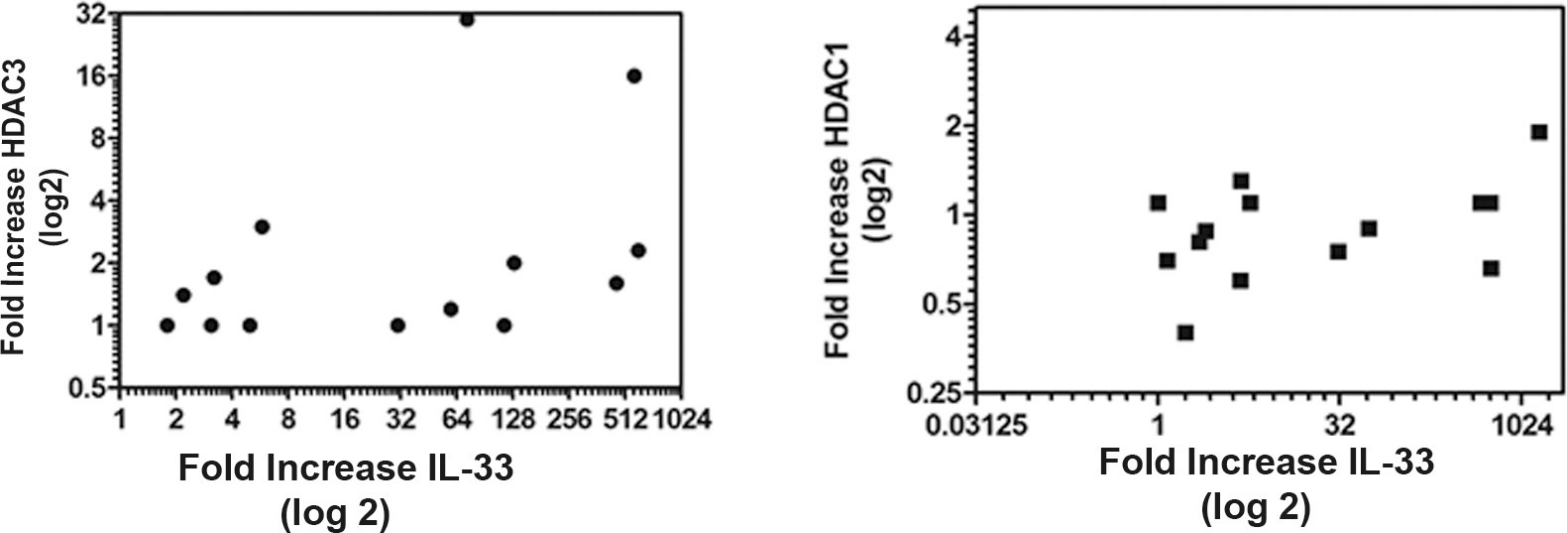
Correlation of IL-33 expression with expression of HDAC3 (left panel) or HDAC1 (right panel). The data are expressed as the Log(2) fold increase of HDAC3 (left panel) to the maximal level of expression of IL-33, correlative statistic of, r = 0.6, p = 0.018 and HDAC1 (right panel). Each symbol represents an individual patient The correlation statistic between expression of HDAC1 and IL-33 was not significant.

We had previously shown increased expression of HDAC3 in MS patients. We had also shown that HDAC3 was a key epigenetic regulator of IL-33 in animal models of asthma. We examined the fold change in maximal expression of HDAC3 in groups 1–3 compared to month 12, which was the time of the lowest expression levels of HDAC3. In fourteen patients, there was a 2.83±5.62 fold increase in the expression of HDAC3 in MS patients. When we correlated the maximal fold increase of IL-33 with the expression of HDAC3, a modest correlation was seen (correlative statistic of, r = 0.6, p = 0.018). We found no correlation between expression of HDAC1 and IL-33 (Fig 5).

### Expression of IL-33 driven downstream genes in MS patients after relapse

**Fig 6:**
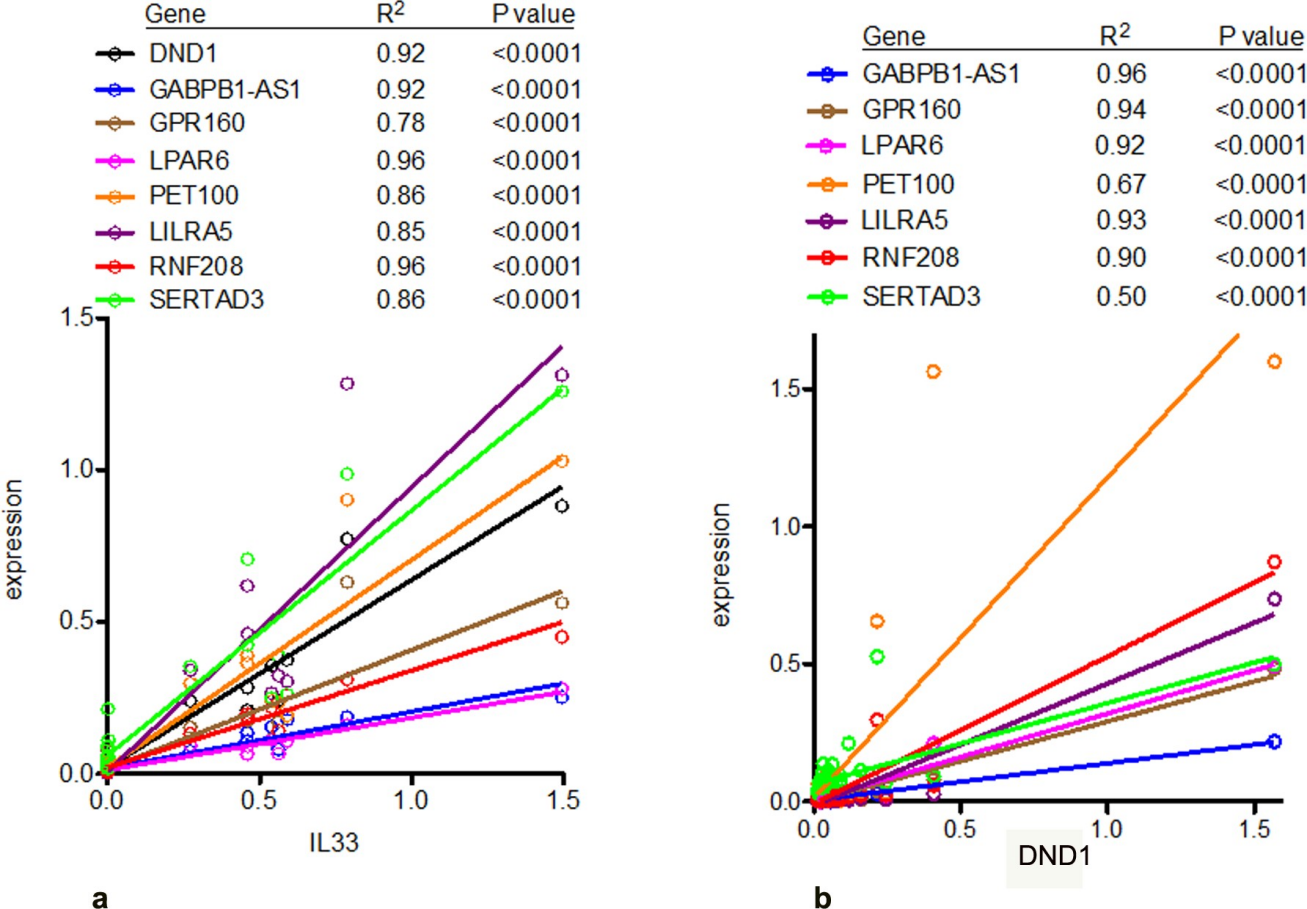
a)IL-33 levels are correlated with expression of multiple genes during MS relapse and remission. mRNA were sampled from the different groups outlined in Fig 2. Expression levels of IL-33 and the indicated genes were determined by PCR and normalized to levels of GAPDH. Correlations were determined by linear regression (R^2^). P-values are the probability that the slope of the line is non-zero. (b) DND1 levels are correlated with expression of multiple genes in patients with CIS. Expression levels of DND1 and the indicated genes were determined by PCR and normalized to levels of GAPDH. Correlations were determined by linear regression (R^2^). P values are the probability that the slope of the line is non-zero.

**Fig 7:**
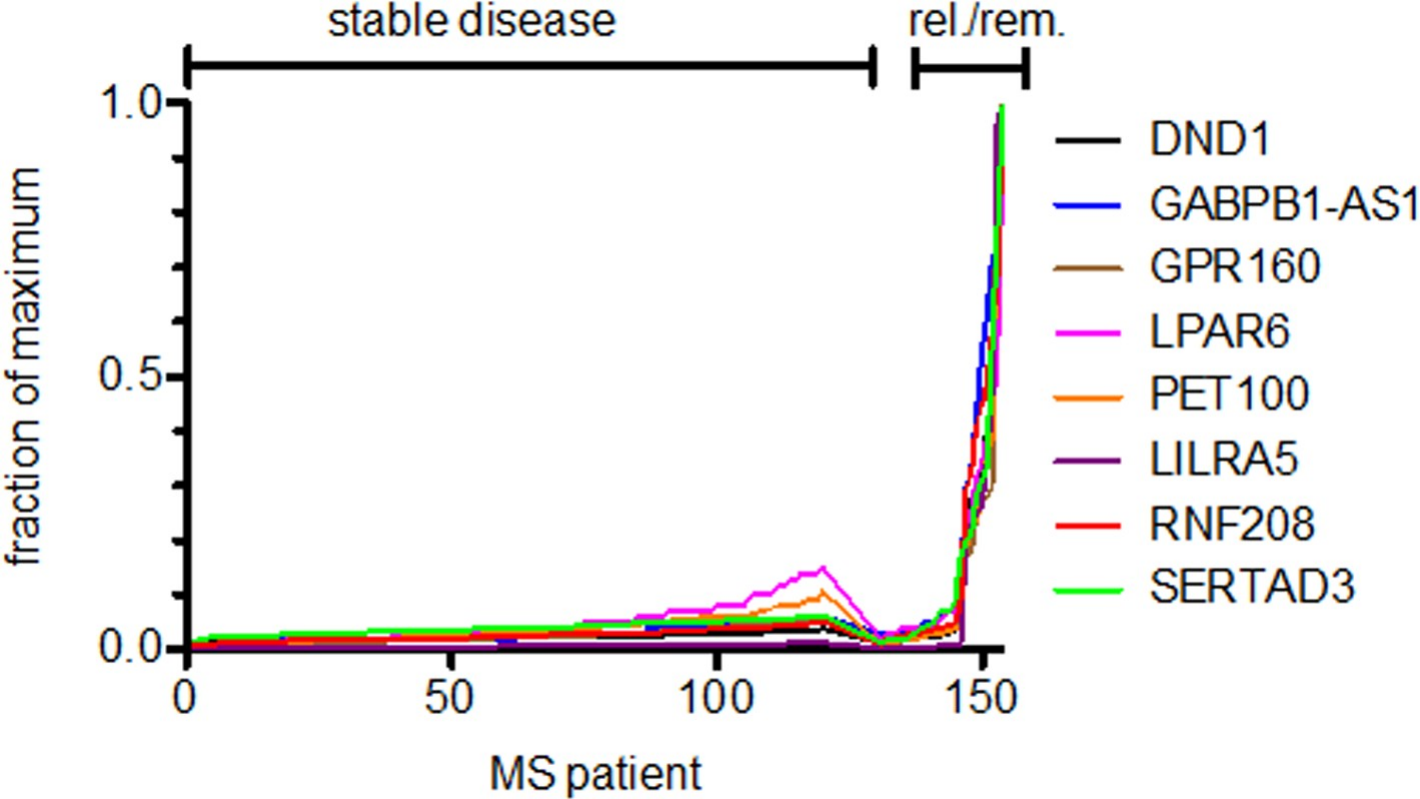
Expression levels of the indicated genes in subjects with MS with stable disease activity compared to those undergoing relapse and remission. Expression levels of the indicated genes were determined by PCR and normalized to GAPDH. Results are expressed as the fraction of the maximum level of expression of each gene. Subjects are grouped into those with stable disease (N = 125) and those undergoing relapse/remission (N = 24).

Our previous studies showed that IL-33 contributed to the shape of the transcriptome in patients presenting with the clinically isolated syndrome event but prior to diagnosis of clinically definite relapsing remitting MS. Therefore, we asked if IL-33 expression levels correlated with expression of target genes identified in prior studies in these patients undergoing relapse and remission. We examined samples from the cohort of RRMS patients in whom we followed and determined IL-33 by qRT-PCR; we randomly selected 3 patients from group 1, 3 from group 2, 4 from group 3 and 3 from group 3 for further analysis. We found that IL-33 expression was highly correlated with multiple protein-coding genes in the relapse-remission cohort (Fig 6A). We confirmed that expression levels of these genes were also highly correlated with expression levels of DND1 in the MS-CIS (patients from Fig 1) cohort with the peak expression of DND1 occurring between 4–7 months after the reported initial attack of CNS demyelination (Fig 6B, note that for these studies we compared expression levels of DND1, Y-axis, to these other genes since we did not determine IL33 levels in these samples due to limiting amounts of RNA). In contrast to the MS-CIS cohort and the cohort of MS patients undergoing relapse and remission, we found that expression of these genes was uniformly low in MS patients with stable disease (Fig 7).

**Fig 8:**
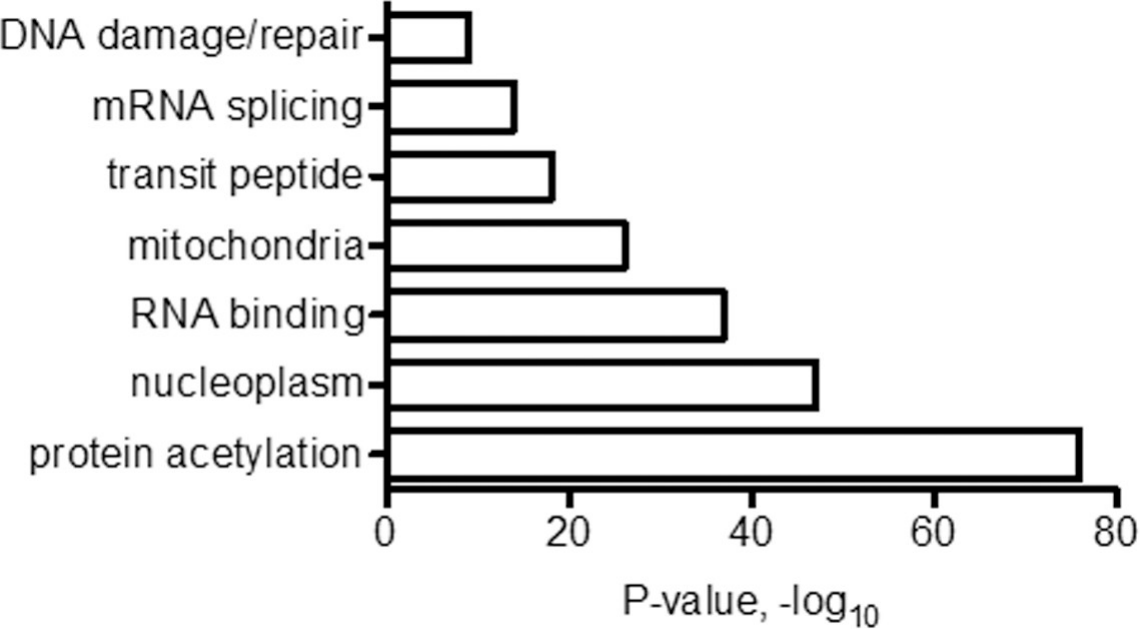
Pathways analysis of genes co-expressed with IL-33 in MS. Whole genome mRNA expression profiles were determined by RNA-seq (HC (N = 8), MS-CIS (N = 6) and MS-naïve (N = 6)) expressed as FPKM. Correlations of expression of DND1, PET100, GPR160, LPAR6, and SERTAD3 to all expressed protein-coding genes were determined. Average correlation coefficients were determined for each protein-coding gene compared to DND1, PET100, GPR160, LPAR6, and SERTAD3. Lists were prepared if r>0.75, P<0.0001.

To gain insight into the potential functional consequences of altered expression of IL-33 and genes which correlated with IL-33 expression, we performed additional analyses using whole genome RNA-sequencing data from MS-CIS (N = 6) and established MS patients (N = 6) (on no treatment at the time of diagnosis) cohorts. We calculated correlation coefficients for each expressed protein-coding gene (N = 12850) and individual genes identified above, DND1, PET100, GPR160, LPAR6, and SERTAD3. We averaged the five correlation coefficients obtained for each expressed protein-coding gene. We generated a gene list that included each gene where the average correlation coefficient was >0.75, P<0.0001 for a total of 1966 genes. We submitted the list to DAVID bioinformatics database to produce a functional annotation chart. Functional categories identified from this gene list included protein acetylation, nucleoplasm, RNA binding, mitochondria, transit peptide, mRNA splicing and DNA damage/repair (Fig 8) S4 Table.

## Discussion

Our study shows changes in the expression of IL-33 during the course of recovery from radiological and clinical relapses in RRMS. We demonstrate that IL-33 expression pattern was dynamic with peak expression levels which varied between MS patients. In the first set of experiments on 22 patients, 18 patients showed an increase in IL-33 6–12 months after relapse. In four patients the highest expression was seen at month 0 at the time of recruitment into the study. Flow cytometry studies further confirmed an increase in expression of IL-33 protein in PBMC following relapse. While the expression of IL-33 increased as recovery ensued, the timeline of clinical recovery and resolution of neurological deficits did not necessarily correlate with maximal IL-33 expression. This is not surprising since it is unlikely that changes in inflammation within the CNS will correlate systemic changes in IL-33.

While it could be argued that increased IL-33 represents a marker of CNS inflammation, we feel that this is not always the case. Highest level of IL-33 at month 0 was seen in four patients, two of whom (Table 1 patients #6 and #10) had new enhancing lesions. However, in the majority of patients, IL-33 levels did not correlate with worsening MRI measures of inflammation. In addition, in two patients (Tables 1, 2 patients #5 and #6) there was a new enhancing MRI lesion at month 12–15 with no concomitant spike in IL-33.

We were also able to see a similar pattern of IL-33 expression in PBMC examined by flow cytometry. Expression levels of IL-33 in all lymphocytes changed during the time following a relapse. In 4 out of 12 RRMS patients, highest expression was seen at month 0 compared to either month 6–9 or 12–14. In 7 patients, the highest expression was seen either between month 6–9 or 12–14 when compared to month 0. The expression levels at the highest time points in RRMS patients were higher than that seen in the OND control group. It is unlikely that differences in IL-33 expression could be accounted by release of IL-33 from the nucleus following a 24 hour culture. As with other alarmins IL-33 is released following death of the cell or in the presence of high extracellular levels of ATP, which is not seen under current culture conditions.

One possibility for the high expression of IL-33 at month 0 in some of the patients could be related to the patients recall of the timing of the onset of the attack. Also, enhancing lesions can remain active for 1–2 months and hence an earlier onset than what the patient reported is a distinct possibility. Our study also shows the relatively short window of time wherein the IL-33 is highly expressed. This would suggest that we could have missed an increase in IL-33 at time points before our second blood draw (month 6) and entry into the study (month 0).

The differences in levels of IL-33 are not likely to be based on the treatment they were receiving, since there were no differences in IL-33 between patients on different DMT’s. Our conclusion from these studies is that while the induction of IL-33 in MS patients is a predictable event, which is independent of therapy, the time kinetics and timing of the response varies between patients. Thus far, the expression of IL-33 either in the acute or recovery phase of MS has not been examined and hence our observations are novel.

One of the surprising aspects of our correlative studies is the relationship between the genes which show high correlation with IL-33. We used our MS-C and MS-N RNA-seq data to identify genes whose expression levels correlated with IL-33 and pathway analysis to identify potential impacted pathways. Of note, this analysis identified RNA binding/mRNA splicing and extensive defects in mRNA processing and splicing to be present in MS [32]. Mitochondrial function is a second process and prior studies show a link between mitochondrial dysfunction and RRMS disease progression as well as during relapse and remission [33,34]. Two of the genes we identified, PET100 and GABPB1-AS1, are directly involved in the biogenesis of cytochrome C oxidase providing a potential link to mitochondrial dysfunction [35]. DNA damage/repair function is a third process and defects in DNA damage pathways exist in RRMS [36]. Finally, mitochondrial damage and dysfunction results in release of ATP which is a strong trigger for IL-33 release. A model whereby mitochondrial damage that occurs during the relapse phase of RRMS may result in release of ATP, which in turn stimulates increased expression of IL-33 to initiate the repair process [15].

In the absence of a known innate immune signal, the factors which induce IL-33 both in the CNS and in PBMC are not known. We had suggested that the induction of IL-33 is likely through activation of epigenetic pathways. Our previous studies have shown an increase in expression of HDAC3 in MS. [37]. IL-33 is expressed at high levels in the CNS and is seen constitutively in astrocytes and oligodendrocytes in rodent brain [13,20]. In addition, in a cross sectional analysis of RRMS patients, expression of IL-33 correlated with HDAC3. HDAC’s act by transcriptional repression and hence the induction of pro-inflammatory gene expression appears at first to be counterintuitive [38,39]. Following stimulation of cells lacking HDAC3 with LPS, there is a loss of expression of over 50% of the inflammatory genes suggesting that, in the normal situation, acetylation keeps inflammatory genes in a repressed state [40]. More recently, HDAC3 has been shown to be necessary to activate IL-4 and loss of HDAC’s limits IL-4 mediated allergic disease [41]. Whether HDAC3 mediates IL-33 expression or not is not known although it is known to activate IL-1.

In MS patients, the period following relapse is associated with tremendous but transient spikes in IL-33. Otherwise, IL-33 levels are relatively low in RRMS as seen in Fig 7. For these reasons we had anticipated genes encoding proteins with functions in either the innate or adaptive immune system to be seen in the course of recovery; that they did not score high in this analysis was surprising. Earlier studies had suggested that polarization of macrophages to the M2 phenotype was an important mechanism by which IL-33 mediated repair in damaged tissue [42,43,44]. However, gene expression profiles failed to show increase in M2 cytokines in MS patients following relapse. At this point, we do not know if IL-33 is the major driver of the repair process or represents one of several pathways involved in the repair process. Notably, we and others have shown that IL-33 promotes recovery and repair in experimental models of CNS demyelination.

Our studies show that a unique family of genes is activated following a relapse. Induction of these genes in PBMC following an inflammatory lesion in the brain suggests a close relationship to the recovery from relapse as evidenced by improvement of MRI measures. Most of these genes have not as yet been described as markers for MS. The function of these genes in MS is speculative but their role in regulation of cellular energy may suggest that identification of these genes will be important in recognizing potential genes which can reflect repair and offer targets for therapy.

## Supporting information

S1 Table PCR primers for the respective genes amplified using q PCR. (DOCX)

S2 Table Demographics of the three patient cohorts involved in the study: RRMS, cohort 1 and cohort 2, CIS patients from the Accelerated cure project. (DOCX)

S3 Table List of genes plated on the TLDA plate. (XLSX)

S4 Table Expression levels of genes within MS-C, MS-N, MS-E and HC cohorts that correlate with expression levels of DND1, PET100, GPR160, LPAR6, and SERTAD3. Columns A-B: gene identification numbers and gene symbols, columns C-AB: expression levels of the indicated genes determined by RNA-seq in FPKM from the indicated cohorts: MS-C, n = 6, MS-N, n = 6, MS-E, n = 6, HC, n = 8. Columns AC-AJ: correlation coefficients of the indicated genes, rows, with DND1 (column AD), PET100 (column AE), GPR160 (column AF), LPAR6 (column AG), SERTAD3 (column AH) and the average correlation coefficient (column AJ). Average expression levels of the indicated genes are also shown (columns AJ-AN) as well as fold difference between MS cohorts and HC cohort, log2 (columns AO-AR) and P values, unpaired T test with Welch’s correction.
(XLSX)

S1 Fig Flow cytometric analysis of a patients PBC subjected to staining with anti IL-33 and anti -CD14 antibodies respectively. The samples were gated using isotype specific antibody as the negative control.
(TIF)
