# Supplementary material for: Longitudinal changes in the expression of IL-33 and IL-33 regulated genes in relapsing remitting MS
Source: PLoS One. 2018 Dec 18;13(12):e0208755. doi: 10.1371/journal.pone.0208755 (PMC6298727; doi:10.1371/journal.pone.0208755)
Supplement: S1 Table — (DOCX) [file pone.0208755.s001.docx]

**Supporting Information**

**S1 Table**

PCR primers for genes amplified using q PCR

**GENE FORWARD PRIMER REVERSE PRIMER**

HDAC1 GAGATGACCAAGTACCACAGC TGACAGAACTCAAACAGGCC

HDAC3 ATTAACTGGGCTGGTGGTC ATGTCAATGTAGAGCACCCG

HDAC4 ACAAGGAGAAGGGCAAAGAG GCGTTTTCCCGTACCAGTAG

HDAC5 ACAAGGAGAAGAGCAAAGAGAG GGAGGGAATGGTTGAGGC

IL33 #3 TTCACCTATTACAGAGTATCTTGCTTC TTTTTCAAGTCTTCAACATATATCTCA
