# Supplementary material for: Longitudinal changes in the expression of IL-33 and IL-33 regulated genes in relapsing remitting MS
Source: PLoS One. 2018 Dec 18;13(12):e0208755. doi: 10.1371/journal.pone.0208755 (PMC6298727; doi:10.1371/journal.pone.0208755)
Supplement: S2 Table — (DOCX) [file pone.0208755.s002.docx]

S2 Table: Summary of demographic of MS patients in the three different

cohorts.

|  | Total Patients | Male | Female | Age | MRI at month 0  New lesions | MRI month 0  No new lesions |
| --- | --- | --- | --- | --- | --- | --- |
| RRMS Cohort 1 | 22 | 9 | 13 | 35.5 | 17 | 5 |
| RRMS Cohort 2 | 12 | 3 | 9 | 38.5 | 10 | 2 |
| CIS cohort | 46 | 13 | 33 | 35.5 | NA | NA |
