# Supplementary figures and images for: Longitudinal changes in the expression of IL-33 and IL-33 regulated genes in relapsing remitting MS
Source: PLoS One. 2018 Dec 18;13(12):e0208755. doi: 10.1371/journal.pone.0208755 (PMC6298727; doi:10.1371/journal.pone.0208755)

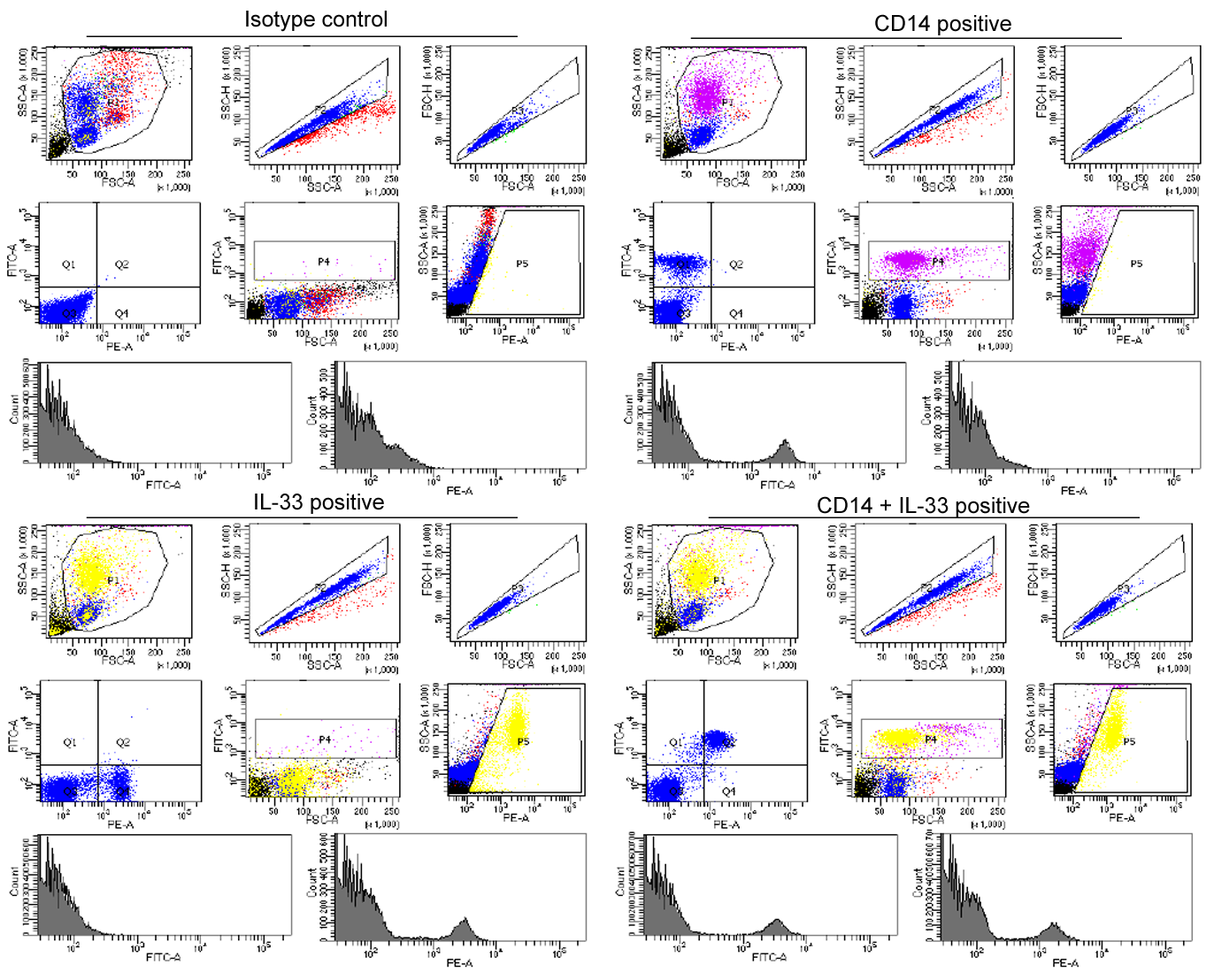

Supplement: S1 Fig — The samples were gated using isotype specific antibody as the negative control. (TIF) [file pone.0208755.s005.tif]
